# Quarterly Report of the Obstetric Practice in the Philadelphia Dispensary

**Published:** 1839-04-13

**Authors:** 

**Affiliations:** Accoucheur


### PHILADELPHIA DISPENSARY.

*Quarterly Report of the Obstetric Practice in the Philadelphia Dispensary.* Dr. Warrington, Accoucheur.

Since the annual report for 1838, thirty-six cases of labour, at term, have been attended to in this institution.

Twenty-one boys, and sixteen girls, have been delivered—one woman having twin daughters.

Of twenty-four cases in which the position of the child was carefully noted, sixteen presented in the first, six in the second, one in the fifth, of the vertex, and one in the first position of the breech.

The average duration of labour, in seventeen cases, was nine hours and forty-two minutes—the extremes being two, and twenty-six hours.

The average time required for the spontaneous delivery of the placenta in twenty-five cases, was twenty-six minutes—the extremes being two, and two hundred and forty minutes.

Several cases occurred, in which, in consequence of the contraction of the os uteri, the placenta was retained, requiring the introduction of the finger, or whole hand, to bring it down edgewise.

In one case, the chorion was found adherent to a considerable portion of the surface of the uterus after the expulsion of the placenta. It was detached by the careful introduction of the hand.

There were two cases of hæmorrhage during labour,—one commencing several days previous to regular uterine contractions, and the other at the beginning of actual labour. The hæmorrhage subsided in both cases as soon as the first stage of labour was completed. In one of these cases, the edge of the placenta could be distinctly felt at the os uteri; in the other it was less satisfactorily recognised.

There was one case of rigidity of the os uteri, which, after irregular, but powerful contractions of the uterus for several days, yielded, very slowly, under the use of free bleeding, purging, and morphia.

In one case, as soon as a very large bag of waters was ruptured, the greater part of the umbilical cord descended into the vagina, and, in consequence of the firmness with which the head immediately engaged in the superior strait, it was impossible to return it. The forceps were applied, and the child delivered in about three minutes after the discovery of the fact. About two inches of the fœtal extremity of the cord pulsated pretty strong when the child was extruded; respiration, however, could not be established, although vigorous efforts were made by my pupil, I. H. Harrisson, and myself, for more than half an hour.

One patient, the subject of the crotchet delivery alluded to in last report, was taken in labour at term, one year and fifteen days after that event. Upon a careful examination, the lower part of the sacrum presented a slight convexity forwards, thus considerably reducing the antero-posterior diameter of the cavity and inferior strait of the pelvis. The first stage of labour was completed in about ten hours; the membranes ruptured spontaneously. The child's head remained wedged at the upper part of the cavity of the pelvis, without advancing under very active uterine contractions. The forceps were applied, and a fine, healthy child, delivered in half an hour, in the presence of several members of the obstetric class. The patient and child did well, the mother resuming her domestic duties at the end of the second week.

The forceps were also applied in a case under the care of Dr. Berkeley, one of the district physicians, in consequence of ineffectual efforts of the uterus to deliver the child from the inferior strait. The object was readily and safely effected.

There were several cases of uterine congestion with great tenderness in the hypogastric region, but generally without fever: all readily yielded to moderate bleeding, oily purgatives, and warm fomentations.

There was one case of well-marked metro-peritonitis; it promptly recovered under the free use of the lancet, and application of leeches to the vulva and hypogastrium.

In addition to the above, there was one case of abortion at the fifth month of gestation, in which the child appeared to have been dead for some time.

A premature delivery at seven months, with the feet presenting; child lived about twelve hours. One at near eight months, presenting the breech in the fourth position; child lived about six hours.

Several children were attacked with ophthalmia; most of them recovered rapidly under the use of solution of nitrate of silver, and mucilage of the medulla sassafras. One child, for whom no nurse could be obtained, suffered from great intensity of the disease, and died in convulsions on the eighth day.

One case of monstrosity occurred in a child, which died in a few days from defect of organization.

Thirty of the above cases of labour occurred in the presence of some one or more members of Dr. Warrington's class.

The following report of a case is furnished by Dr. Patterson, one of the district physicians for the dispensary:

*Feb. 16th*, 1839.—I. N. had irregular pains; os uteri not dilated.

*17th.*—Pains continued all night, now more frequent and severe; os uteri still closed. Attempted bleeding, but succeeded in getting only ℥v. to ℥vj.

*Evening.*—Os uteri dilated about a line in diameter. Bled ℥xxv.

*18th.*—Pains frequent and forcing; os uteri size of half a dime. Called Dr. Warrington. Bled ℥xxx. Gave tart. antim. freely. It caused vomiting, but not much relaxation.

*Evening.*—The distended bladder formed a prominent and distinct tumour in the hypogastrium. With some difficulty a very small male catheter was introduced, and more than a quart of urine drawn off. Bled from a vein in each arm, the patient standing; but, although syncope did not ensue, could obtain only about ℥v. of blood.

*Evening*, 11 *o'clock.*—Os uteri still undilated beyond the size of a dime. The waters had passed off more than twenty-four hours since. The suggillated scalp could be felt forming a tumour protruding through the small opening in the uterus. Made a free application of softened extract of belladonna to the os and cervix uteri. Dilatation now proceeded so rapidly, that the child was born in the presence of a medical friend, at 2 A. M., of the 19th. The child was dead, and the scalp greatly ecchymosed. The uterus remained in a state of congestion for several days afterwards. Patient recovered.

---

*University of Pennsylvania.*—The medical class in this institution for the session 1838–9, numbered *four hundred and two* students.

At a public commencement, held April 5th, 1839, the degree of Doctor of Medicine was conferred by the Rev. Provost, John Ludlow, D. D. on *one hundred and forty-six* gentlemen. An eloquent and impressive charge was delivered by Professor Chapman.

---

*To the Editors of the Medical Examiner.*

Gentlemen,—Enclosed is a general statistic of the cases treated in the Wills Hospital since its opening in March, 1834, down to January 1, 1839. Should it be appropriate to the pages of your journal, it is at your disposal.

Respectfully,

Jno. Neill, Resident.

*Wills Hospital, April* 12, 1839.

